# Predictors of First-Pass Effect in Endovascular Thrombectomy With Stent-Retriever Devices for Acute Large Vessel Occlusion Stroke

**DOI:** 10.3389/fneur.2022.664140

**Published:** 2022-03-25

**Authors:** Chu Chen, Tangqin Zhang, Youqing Xu, Xiangjun Xu, Junfeng Xu, Ke Yang, Lili Yuan, Qian Yang, Xianjun Huang, Zhiming Zhou

**Affiliations:** Department of Neurology, Yijishan Hospital, Wannan Medical College, Wuhu, China

**Keywords:** endovascular thrombectomy, stroke, stent-retriever, first-pass effect, recanalization

## Abstract

**Background and Purpose:**

Successful recanalization after the first pass of the device in endovascular thrombectomy (EVT) can significantly improve patients' prognosis. We aimed to investigate the possible factors that influence achieving the first-pass effect (FPE).

**Methods:**

We retrospectively analyzed the patients who underwent EVT caused by anterior circulation large vessel occlusion stroke (ALVOS) in our center. The FPE was defined as a successful recanalization [modified Thrombolysis in Cerebral Infarction (mTICI) 2b/3 defined as modified FPE (mFPE); mTICI 3 as true FPE (tFPE)] after one pass of the device without rescue therapy. Univariate and multivariate regression analyses were used to explore the predictors of FPE and the relationship between FPE and prognosis.

**Results:**

There were 278 patients (age, 69.3 ± 10.9 years, male, 51.1%) included, 30.2% of them achieved mFPE, while 21.2% achieved tFPE. We found the higher clot burden score (CBS), the truncal-type occlusion, and the favorable anatomy of both extracranial and intracranial segments of the internal carotid artery (ICA) were associated with achieving mFPE. The higher CBS and truncal-type occlusion were statistically significant predictors of tFPE. Moreover, FPE was significantly associated with improved clinical outcomes, regardless of mFPE and tFPE.

**Conclusions:**

The CBS, tortuosity of ICA, and angiographic occlusion type were independent predictors of achieving FPE. The rate of improved clinical and safety outcomes was higher in patients with FPE, which has important clinical significance.

## Introduction

Endovascular thrombectomy (EVT) with stent-retriever (SR) has been recommended as a first-line treatment for patients with acute large vessel occlusion stroke (ALVOS) (1). However, in the real world, to achieve complete recanalization, multiple attempts and rescue therapy were often required, which may prolong the procedure time and cause vascular endothelial injury, leading to a high incidence of hemorrhagic transformations (2).

Previous studies suggest that patients had significantly improved outcomes with successful recanalization after the first pass of the thrombectomy device (3). Hence, a new conception has been proposed: the “true first-pass effect” (tFPE), defined as complete revascularization (modified Thrombolysis in Cerebral Infarction, mTICI 3) after one single pass of the device without rescue therapy (4). Modified first-pass effect (mFPE) was defined as mTICI 2b/3 after one pass of the device (3). The first-pass effect may therefore constitute the main goal in the treatment of patients with ALVOS in the EVT era.

Current evidence demonstrated an association between procedural difficulty and angiographic occlusion type (5). Moreover, the tortuosity of the vascular anatomy can also present technical challenges during the procedure (6, 7). However, the factors that affected the achievement of mFPE have not yet been clear. We aimed to investigate the possible factors that influence achieving mFPE in patients undergoing EVT with stent-retriever devices.

## Methods

### Patients and Baseline Variables

We enrolled patients treated with emergency EVT in Yijishan Hospital, Wannan Medical College from July 2015 to June 2020. The local ethics committee approved the study protocol. Patients were included in our study if they were treated with stent-retriever devices (Solitaire, Covidien, Irvine, California, USA). Time from onset to puncture, baseline modified Rankin Scale (mRS) score, National Institute of Health Stroke Scale (NIHSS) score, and Alberta Stroke Program Early CT (ASPECT) score were not considered as exclusion criteria.

All consecutive patients were prospectively documented. This included demographics, medical history (hypertension, diabetes mellitus), NIHSS score, the Trial of ORG 10172 in Acute Stroke Treatment (TOAST) classification, laboratory measures, procedural factors (ASPECT score, leptomeningeal collaterals, and occlusion site), and outcomes. For all enrolled subjects, the imaging characteristics were evaluated by two neurologists/interventionalists who were blinded to the clinical information.

The proximal middle cerebral artery (MCA) or the anterior cerebral artery (ACA) occlusion was defined as M1 or A1 of MCA/ACA occlusion as confirmed by preoperative imaging. The distal MCA/ACA occlusion was M2/A2 or distal of M2/A2 MCA/ACA occlusion.

### True First-Pass Effect

According to previous studies, the tFPE was defined as follows: (1) one single pass of the device; (2) near-complete revascularization of the large vessel occlusion and its downstream territory (mTICI 3); (3) with no use of rescue therapy (4).

### Modified First-Pass Effect

The mFPE was defined as follows: (1) one single pass of the device; (2) near-complete revascularization of the large vessel occlusion and its downstream territory (mTICI 2b/3); (3) with no use of rescue therapy (3, 8).

### Collateral Circulation State

Collateral circulation was assessed based on retrograde contrast opacification of vessels within the occluded territory on delayed digital subtraction angiography (DSA) images. The collateral state was classified as follows: grade 0 was assigned if there was little or no significant reconstitution in the territory of the occluded vessel or less than one-third of the occluded territory, grade 1 was assigned if the collaterals reached less than two-thirds of the occluded territory, and grade 2 was assigned if the collateralization reached more than two-thirds of the territory or the proximal main stem (9).

### Clot Burden Score

The clot burden score (CBS) is an angiography-based scoring system that calculates a score from 0 to 10 to determine the extent of thrombus. Particularly, a score of 10 implies clot absence, while a score of 0 means that the vessel is completely occluded. A score of 2 is subtracted if the thrombus is found in each of the supraclinoid internal carotid artery (ICA), the proximal half of the MCA trunk, and the distal half of MCA trunk. A score of 1 is subtracted if the thrombus is found in the infraclinoid ICA, ACA, and for each affected MCA M2 branch (10).

### Angiographic Occlusion Type Classification

**Figure 1:**
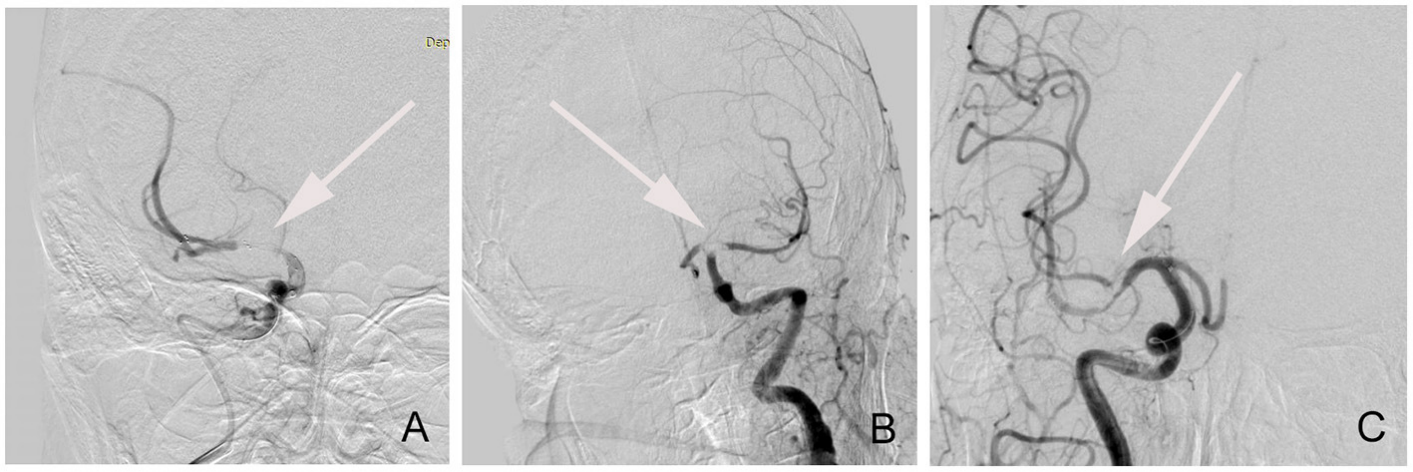
The classification of angiographic occlusion type. **(A)** Truncal-type occlusion; **(B)** T-shaped branching-site occlusion; **(C)** Y-shaped branching-site occlusion.

For all enrolled patients with ALOVS, the occlusion type was classified as truncal-type or branching-site occlusion through angiography (Figure 1). The occlusion was defined as a branching-site occlusion when (1) contralateral ICA flow could not further advance across the relevant ICA bifurcation site, (2) Y- or T-shaped filling defect involving branching site is directly observed, or (3) another branch could not be seen even on stent-retriever deployment to one branch across occlusion site. Conversely, the occlusion type was classified as truncal-type occlusion when all major branches and relevant bifurcations are clearly observed (5, 11).

### Vascular Tortuosity Classification and Measurements

The anatomy may exist alone or in combination at many different levels, including the ICA and aortic arch. We divided the ICA vessel into extracranial and intracranial segments to be separately considered. Each type of arterial tortuosity was defined based on prior studies.

**Figure 2:**
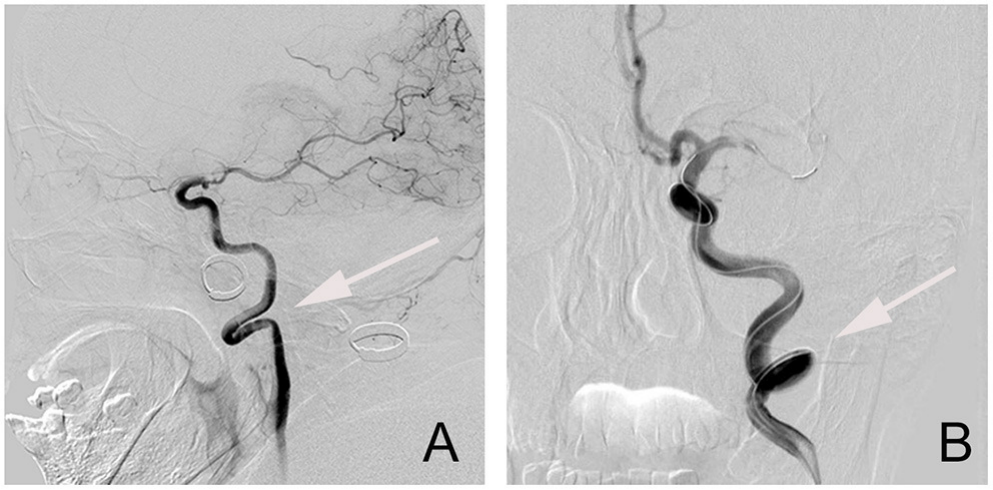
The types of tortuosities of an extracranial segment of the internal carotid artery (ICA) vessel. **(A)** Kinking; **(B)** Coiling.

The extracranial ICA vessel was considered to be straight if the angle between the common carotid artery (CCA) and the ICA centerlines was <15°. Patients were considered to have significant extracranial ICA tortuosity if they were presented with kinking or coiling (12). Kinking represents solitary bends in the ICA with acute (<90°) angulation, while coiling produces a full 360°turn in the artery (13, 14) (Figure 2). We classified the normal vessel or mild tortuosity as grade 1, while severe tortuosity of the vessel, including kinking and coiling, was classified as grade 2.

**Figure 3:**
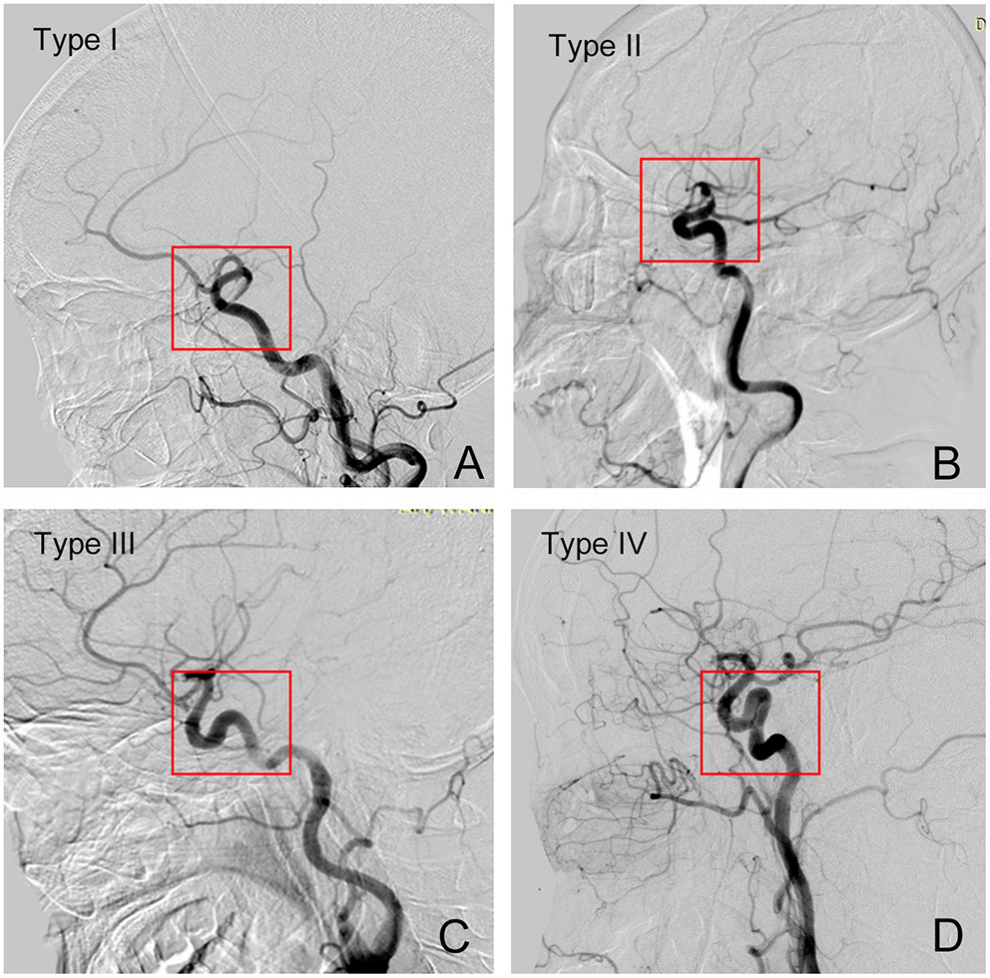
Grades of tortuosity of intercranial segment of the ICA vessel. **(A)** Type I; **(B)** Type II; **(C)** Type III; **(D)** Type IV.

The previous study showed a four-level classification system of the tortuosity of the intracranial ICA segment based on the angular and height differences between the anterior and posterior genera from the top of the posterior genus to the anterior genus. Type I was defined as the anterior and posterior genu of ICA cavernous segment configurations are opened the angle is <90°, while Type II has a more acute angle of anterior genu in comparison to type I. Type III has a superior deflection of posterior genu. Type IV is the most tortuous, with a shape characteristic of Simmons catheter (13, 15) (Figure 3). In the present study, we regarded all types as 3 different grade levels, in which type I was regarded as grade 1, type II/III as grade 2, and type IV as grade 3.

As to aortic arch anatomy, the I, II, and III arch types were determined by the vertical distance from the origin of the innominate artery to the top of the arch. The bovine aortic arch is defined as the common origin of the innominate artery and left CCA or a left CCA that arises directly from the innominate artery (6). We regarded arch type I/II as grade 1 and type III/ bovine aortic arch as grade 2.

### Statistical Analysis

Patients were divided into the mFPE group and non-mFPE group, or good and poor outcomes groups. Continuous variables (age, baseline systolic blood pressure, baseline diastolic blood pressure, NIHSS, ASPECT score, CBS, and procedure time) are presented as the mean ± SD or as the median (IQR). Categorical variables (gender, hypertension, smoke, TOAST, tortuosity of extracranial and intracranial segments of ICA, aortic arch classification, occluded site, collateral state, and angiographic occlusion type) are presented as percentages. Continuous variables were analyzed using the Mann-Whitney U test. Categorical variables were analyzed using the χ^2^ test and the Fisher exact test, as appropriate. We then implemented backward stepwise logistic regression analysis to determine the independent factors of mFPE. In addition, the association between mFPE and clinical outcome or mortality at 90 days was also analyzed. Variables with a value of *p* < 0.1 from the univariate analysis were entered into the logistic regression analysis. ORs and 95% CIs were calculated. For all analyses, a two-tailed value of *p* ≤ 0.05 was considered significant. All statistical analyses were performed using the SPSS (IBM Corp, Armonk, NY. 25.0) software package.

## Results

### Baseline Characteristics

From July 2015 to June 2020, a total of 419 patients who underwent EVT met the inclusion criteria for this study. Of them, 137 were not treated with stent-retriever devices and 4 missed the procedure images and were therefore excluded from our study.

The mean age of the 278 included patients was 69.3 ± 10.9 years, 51.1% (*n* = 142) of which were men, while the median baseline ASPECT score was 8 (IQR 8–10), and the median baseline NIHSS score was 15 (IQR 12–19). Seventeen-point-six percent (*n* = 49) of the patients were identified to have a type III/Bovine configurations aortic arch and 52.2% (*n* = 145) had a severe tortuosity of extracranial ICA. The angiographic occlusion type was determined to be a truncal-type occlusion in 57.6% (*n* = 159) of patients. The other baseline characteristics are shown in Table 1. All eligible patients underwent a 90-day follow-up, 43.9% (*n* = 122) of patients had 90-day functional independence, and 27.0% (*n* = 75) of them died (**Table 3**).

**Table 1:** Univariable analysis of predictors of modified first-pass effect (mFPE) and true first-pass effect (tFPE).

	**All** ***N* = 278**	**mFPE**			**tFPE**		
		**mFPE (+)**	**mFPE (–)**	***P***	**tFPE (+)**	**tFPE (–)**	***P***
		***N* = 84**	***N* = 194**		***N* = 59**	***N* = 219**	
Age, y, mean (SD)	69.3 (10.9)	68.9 (10.1)	69.5 (11.3)	0.674	70 (9.5)	69.1 (11.3)	0.680
Sex (male), *n* (%)	142 (51.1)	51 (60.7)	91 (46.9)	**0.034**	37 (62.7)	105 (47.9)	**0.044**
Smoke, *n* (%)	72 (26.3)	27 (32.9)	45 (23.4)	0.102	20 (34.5)	52 (24.1)	0.110
Hypertension, *n* (%)	202 (72.7)	65 (77.4)	137 (70.6)	0.245	46 (78.0)	156 (71.2)	0.303
Diabetes mellitus, *n* (%)	44 (15.8)	12 (14.3)	32 (16.5)	0.643	10 (16.9)	34 (15.5)	0.790
NIHSS, media (IQR)	15 (12, 19)	15 (12, 19)	16 (13, 19)	0.330	15 (12, 19)	16 (13, 19)	0.397
ASPECT, media (IQR)	8 (8, 10)	8 (8, 10)	8 (8, 9)	0.207	8 (8, 10)	8 (8, 10)	0.263
TOAST, *n* (%)				**0.017**			**0.009**
Atherosclerosis	74 (26.6)	32 (38.1)	42 (21.6)		23 (39.0)	51 (23.3)	
Cardioembolic	175 (62.9)	45 (53.6)	130 (67.0)		32 (54.2)	143 (65.3)	
Other or unknown	29 (10.5)	7 (8.4)	22 (11.3)		4 (6.8)	25 (11.4)	
CBS, media (IQR)	8 (6, 8)	8 (8, 8)	7 (5, 8)	**<0.001**	8 (8, 8)	7 (5, 8)	**<0.001**
Favorable collateral Status, *n* (%)	109 (39.2)	39 (46.4)	70 (36.1)	0.268	30 (50.8)	79 (36.1)	0.115
IVT, *n* (%)	29 (10.4)	7 (8.3)	22 (11.3)	0.451	2 (3.4)	27 (12.3)	**0.046**
Aortic arch, *n* (%)				0.151			0.538
I/II	229 (82.4)	65 (77.4)	164 (84.5)		47 (79.7)	182 (83.1)	
III/Bovine	49 (17.6)	19 (22.6)	30 (15.5)		12 (20.3)	37 (16.9)	
Extracranial segment of ICA, *n* (%)				**0.002**			0.161
Normal or mild tortuosity	133 (47.8)	52 (61.9)	81 (41.8)		33 (55.9)	100 (45.7)	
Severe tortuosity	145 (52.2)	32 (38.1)	113 (58.2)		26 (44.1)	119 (54.3)	
Intracranial segment of ICA, *n* (%)				**0.055**			**0.043**
Type I	176 (63.8)	61 (72.6)	115 (59.9)		43 (72.9)	133 (61.3)	
Type II/III	81 (29.3)	21 (25.0)	60 (31.3)		16 (27.1)	65 (30.0)	
Type IV	19 (6.9)	2 (2.4)	17 (8.9)		0 (0)	19 (8.8)	
Angiographic occlusion type, *n* (%)				**<0.001**			**<0.001**
Truncal-type occlusion	159 (57.6)	68 (81.0)	91 (47.4)		49 (83.1)	110 (50.7)	
Branching-site occlusion	117 (42.4)	16 (19.0)	101 (52.6)		10 (16.9)	107 (49.3)	
Intermediate catheter, *n* (%)	57 (20.5)	24 (28.6)	33 (17.0)	**0.028**	16 (27.1)	41 (18.7)	0.156
Site of occlusion, *n* (%)				**0.013**			0.118
ICA	102 (36.7)	20 (23.8)	82 (42.3)		15 (25.4)	87 (39.7)	
Proximal MCA/ACA	155 (55.7)	56 (66.7)	99 (51.0)		38 (64.4)	117 (53.4)	
Distal MCA/ACA	21 (7.6)	8 (9.5)	13 (6.7)		6 (10.2)	15 (6.8)	

*mFPE, modified first-pass effect; tFPE, true first-pass effect; NIHSS, National Institutes of Health Stroke Scale; ASPECT, Alberta Stroke Program Early CT; TOAST, the Trial of ORG 10172 in Acute Stroke Treatment. IVT, intravenous thrombolysis; CBS, clot burden score; MCA, middle cerebral artery; ACA, anterior cerebral artery; ICA, internal carotid artery; mTICI, modified Thrombolysis in Cerebral Infarction. The bold values indicate the variable's P value < 0.05*.

### Frequency and Predictors of FPE

Among the enrolled patients, mFPE was achieved in 30.2% (84 out of 278) patients. In the mFPE group, a significantly high proportion of male patients (60.7 vs. 46.9%, *p* = 0.034) were present. In addition, patients with mFPE had higher CBS (media, 8 vs. 7; *p* < 0.001) and more patients had mild tortuosity of both extracranial segment (61.9 vs. 41.8%, *p* = 0.002) and intracranial segment of ICA (type I: 72.6 vs. 59.9%, *p* = 0.055). Patients with mFPE were more likely to have a truncal-type occlusion (81.0 vs. 47.4%, *p* < 0.001) and higher rate of atherosclerosis (38.1 vs. 21.6%, *p* = 0.017). In addition, the intermediate catheters were used more frequently (28.6 vs. 17.0%, *p* = 0.028) than the non-mFPE group (Table 1).

After adjustment for confounding factors (sex, TOAST, CBS, the tortuosity of extracranial and intracranial ICA, the occlusion type, the use of intermediate catheter, and the occluded site), we found that lower CBS (OR = 1.384; 95%CI:1.119–1.713; *p* = 0.003) can affect the rate of mFPE. Furthermore, the severe tortuosity of extracranial segment of ICA (OR = 0.506; 95% CI: 0.288–0.889; *p* = 0.018), the Type IV tortuosity of intracranial segment of ICA (Type IV vs. Type I: OR: 0.195, 95% CI: 0.041–0.918, *p* = 0.039), and branching-site occlusion type (OR = 0.354; 95%CI: 0.179–0.701; *p* = 0.003) were also associated with a reduced likelihood of mFPE (Table 2).

**Table 2:** Multivariable analysis of predictors of mFPE and FPE.

	**OR (95%CI)**	***P***
**mFPE**		
CBS	1.384 (1.119–1.713)	0.003
Extracranial segment of ICA	0.506 (0.288–0.889)	0.018
Intracranial segment of ICA		0.112
Type II/III to Type I	0.835 (0.438–1.591)	0.583
Type IV to Type I	0.195 (0.041–0.918)	0.039
Branching-site occlusion	0.354 (0.179–0.701)	0.003
**tFPE**		
CBS	1.335 (1.043–1.709)	0.022
IVT	0.248 (0.054–1.129)	0.071
Branching-site occlusion	0.356 (0.153–0.828)	0.016
TOAST		0.110
Cardioembolic to atherosclerosis	0.574 (0.288–1.142)	0.114
Other or unknown to atherosclerosis	0.305 (0.087–1.067)	0.063

*mFPE, modified first-pass effect; tFPE, true first-pass effect; CBS, clot burden score*.

In addition, tFPE was achieved in 21.2% (59 out of 278) of patients, the details of which are shown in Table 1. When comparing patients' characteristics after adjustment for confounding factors in the backward stepwise logistic regression analysis, we found that higher CBS (OR = 1.328; 95%CI:1.045–1.688; *p* = 0.021) and truncal-type occlusion (OR: 0.356; 95%CI: 0.153–0.828; *p* = 0.016) were statistically significant predictors of tFPE (Table 2).

### FPE and Functional Outcomes

**Figure 4:**
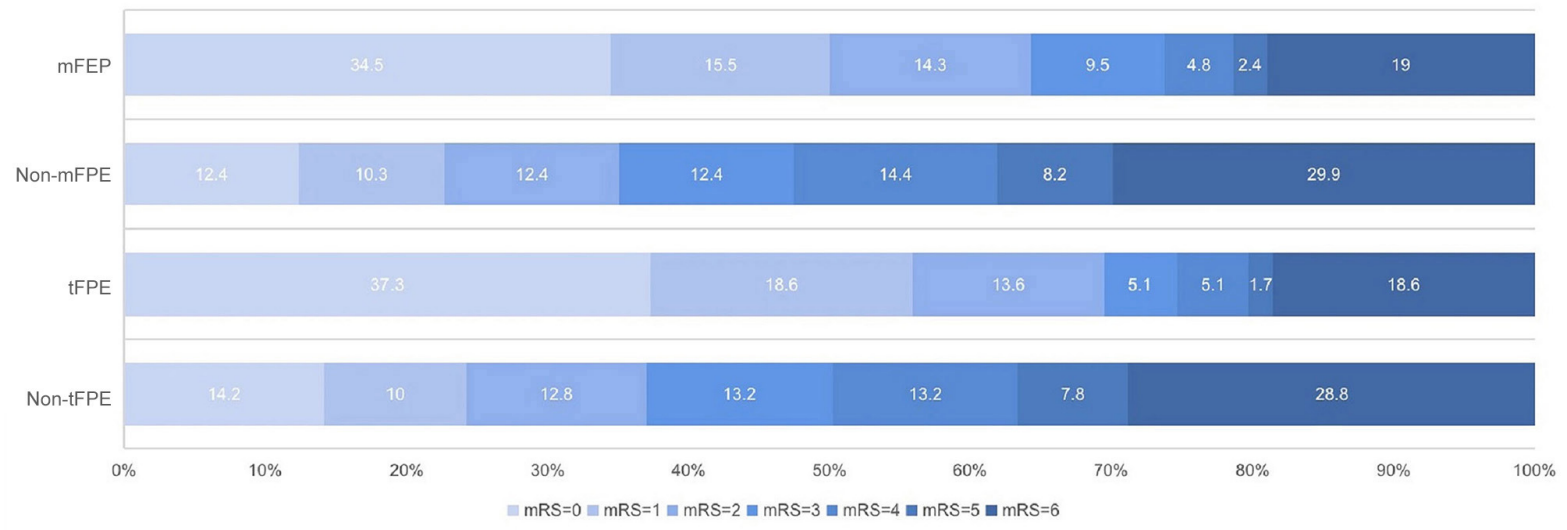
The 90-day modified Rankin Scale (mRS) score in patients who underwent endovascular treatment with or without first-pass effect.

Table 3 demonstrates a significantly shorter median time to revascularization in the mFPE group (media, 40 vs. 78 min, *p* < 0.001). The FPE group of patients had significantly better 90-day clinical outcomes than those in the non-FPE group (Figure 4).

**Table 3:** Comparison in efficacy and safety outcomes according to mFPE and tFPE.

	**All** ***N* = 278**	**mFPE**			**tFPE**		
		**mFPE (+)**	**mFPE (–)**	***P***	**tFPE (+)**	**tFPE (–)**	***P***
		***N* = 84**	***N* = 194**		***N* = 59**	***N* = 219**	
Procedural time, media (IQR)	65 (46, 95)	40 (34, 51)	78 (60, 105)	<0.001	40 (35, 52)	74 (56, 102)	<0.001
Number of passes, media (IQR)	2 (1, 3)	1 (1, 1)	3 (2, 3)	<0.001	1 (1, 1)	2 (2, 3)	<0.001
NIHSS at 24 h, media (IQR)	13 (10, 20)	10 (6, 18)	14 (10, 22)	<0.001	10 (6, 15)	14 (10, 22)	<0.001
sICH, *n* (%)	28 (10.2)	6 (7.1)	22 (11.6)	0.264	2 (3.4)	26 (12.1)	0.051
90d mRS ≤ 2, *n* (%)	122 (43.9)	54 (64.3)	68 (35.1)	<0.001	41 (69.5)	81 (36.0)	<0.001
90 d Death, *n* (%)	75 (27.0)	17 (20.2)	58 (29.9)	0.096	12 (20.3)	63 (28.8)	0.195

*mFPE, modified first-pass effect; tFPE, true first-pass effect; NIHSS, National Institutes of Health Stroke Scale; sICH, Symptomatic Intracerebral Hemorrhage Risk Score; mRS, modified Rankin Scale*.

Based on patients' 90-day functional outcomes, we further divided the population into a good outcome group (mRS ≤ 2) and poor outcome group (mRS > 2), and significant variables in the univariate analysis were entered into the multivariate logistic model (Table 4). After adjusting for variables, including mFPE, age, sex, smoking, NIHSS, ASPECT, TOAST, CBS, collateral, occluded site, mTICI, and sICH, mFPE (OR: 0.347; 95%CI: 0.159–0.760; *p* = 0.008) and tFPE (OR: 0.315; 95%CI: 0.134–0.743; *p* = 0.008), still remained a separate independent predictor of favorable clinical outcome (Table 5).

**Table 4:** Univariable analysis of 90-day outcomes in patients with endovascular thrombectomy (EVT).

	**Good outcome**,	**Poor outcome**,	***P***
	***N* = 122**	***N* = 156**	
Age y, mean (SD)	66.4 (10.9)	71.6 (10.5)	**<0.001**
Sex (male), *n* (%)	71 (58.2)	71 (45.5)	**0.040**
Smoking, *n* (%)	40 (33.3)	32 (20.8)	**0.019**
Baseline SBP, media (IQR)	145 (131, 160)	150 (135.5, 165)	0.111
Baseline DBP, media (IQR)	82 (72, 90)	81 (74, 92)	0.535
HBP, *n* (%)	82 (67.2)	120 (76.9)	0.071
DM, *n* (%)	15 (12.3)	29 (18.6)	0.186
NIHSS, media (IQR)	14 (12, 17)	17 (14, 20)	**<0.001**
ASPECT, media (IQR)	9 (8, 10)	8 (7, 9)	**<0.001**
TOAST, *n* (%)			**<0.001**
Atheroma	46 (37.7)	28 (17.9)	
Cardio-emboli	63 (51.6)	112 (71.8)	
Others	13 (10.6)	16 (10.3)	
OTP, media (IQR)	270 (220, 385)	270 (210, 311)	0.904
OTR, media (IQR)	330 (277, 385)	348 (290, 395)	0.149
CBS, media (IQR)	8 (7, 8)	7 (5, 8)	**<0.001**
Collateral, *n* (%)			**<0.001**
Grade 0	7 (5.7)	61 (39.1)	
Grade 1	42 (34.4)	59 (37.8)	
Grade 2	73 (59.8)	36 (23.1)	
IVT, *n* (%)	15 (12.3)	14 (9.0)	0.431
Modified First-pass effect, *n* (%)	54 (44.3)	30 (19.2)	**<0.001**
True First-pass effect, *n* (%)	41 (33.6)	18 (11.5)	**<0.001**
mTICI <2b, *n* (%)	15 (12.3)	61 (39.1)	**<0.001**
Site of occlusion, *n* (%)			**0.005**
ICA	32 (26.2)	70 (44.9)	
Proximal MCA/ACA	78 (63.9)	77 (49.4)	
Distal MCA/ACA	12 (9.8)	9 (5.8)	
sICH, *n* (%)	2 (1.6)	26 (17.1)	**<0.001**

*NIHSS, National Institutes of Health Stroke Scale; ASPECT, Alberta Stroke Program Early CT; TOAST, the Trial of ORG 10172 in Acute Stroke Treatment. IVT, intravenous thrombolysis; MCA, middle cerebral artery; ACA, anterior cerebral artery; ICA, internal carotid artery; mTICI, modified Thrombolysis in Cerebral Infarction; OTP, symptom onset to groin puncture time; OTR, time from stroke onset to recanalization; baseline SBP, baseline systolic blood pressure; baseline DBP, baseline diastolic blood pressure; sICH, Symptomatic Intracerebral Hemorrhage Risk Score. The bold values indicate the variable's P value < 0.05*.

**Table 5:** Multivariable analysis of predictors of 90-day outcomes mFPE and FPE.

	**OR (95%CI)**	***P***
**mFPE**		
Age	1.038 (1.007–1.070)	0.016
NIHSS	1.133 (1.042–1.231)	0.003
ASPECT	0.639 (0.484–0.843)	0.002
CBS	0.826 (0.683–0.999)	0.049
Collateral		<0.001
Grade 1 to Grade 0	11.351 (3.860–33.378)	<0.001
Grade 2 to Grade 0	2.396 (1.211–4.741)	0.012
Modified First-pass effect	0.347 (0.159–0.760)	0.008
mTICI <2b	0.486 (0.211–1.118)	0.090
sICH	8.553 (1.535–47.655)	0.014
**tFPE**		
Age	1.043 (1.011–1.076)	0.008
NIHSS	1.130 (1.041–1.228)	0.004
ASPECT	0.643 (0.488–0.848)	0.002
CBS	0.806 (0.668–0.973)	0.025
Collateral		<0.001
Grade 1 to Grade 0	10.220 (3.571–29.249)	<0.001
Grade 2 to Grade 0	2.338 (1.179–4.639)	0.015
True First-pass effect	0.315 (0.134–0.743)	0.008
mTICI <2b	0.439 (0.195–0.990)	0.047
sICH	7.904 (1.509–41.391)	0.014

*mFPE, modified first-pass effect; tFPE, true first-pass effect; NIHSS, National Institutes of Health Stroke Scale; ASPECT, Alberta Stroke Program Early CT; CBS, clot burden score; mTICI, modified Thrombolysis in Cerebral Infarction; sICH, Symptomatic Intracerebral Hemorrhage Risk Score*.

## Discussion

In our study, the predictors that reduced reaching FPE include lower CBS and branching-site occlusion type. However, the severe tortuous anatomy of both extracranial and intracranial segments of ICA could reduce reaching mFPE. Patients who achieved FPE, regardless of mFPE and tFPE, had shorter procedure time, lower NIHSS score at 24 h, and were associated with a higher likelihood of favorable outcomes.

The frequency of FPE in our study was 30.2% (mFPE) and 21.2% (tFPE). In general, the probability of achieving mFPE in previous studies was 39–47.3% (16, 17), while tFPE was 25.1–38.7% (4, 8). The difference in frequency among these studies may be due to their combined strategies and the use of different thrombectomy devices.

In this study, we found that the tortuosity of the extracranial segment of ICA can significantly affect the acquisition of mFPE (severe tortuosity vs. normal or mild tortuosity: OR = 0.506; 95%CI: 0.288–0.889; *p* = 0.018). Moreover, in the intracranial segment of ICA, the mFPE rate of Type I is five times more than Type IV (Type IV vs. Type I: OR: 0.195, 95%CI: 0.041–0.918, *p* = 0.039). Tortuous vascular pathways may be age-related degenerative changes and associated with the burden of atherosclerotic disease (18). A previous histological examination of tortuous ICAs found that metaplastic changes occurred in the tunica media of affected vessels, wherein muscular and elastic tissue was replaced by loose connective tissue (19). The severe anatomic difficulties could not only affect the ability and speed of SR getting into the target vessel, but also hinder the removal of the thrombus. The stent may get stuck in the curved vessels and be stretched under the pulling force, deforming the stent that originally fit closely with thrombi, resulting in detachment (7). However, with the development of technology and equipment of EVT, the newer-generation large-bore contact aspiration (AC) or distal access catheter (DAC) was suggested to be used to overcome the tortuosity of the vascular path to provide a good support for distal progression and aspiration capacity (20). In addition, it is suggested that a direct carotid approach should be considered when a patient is presented with unfavorable anatomy to minimize the operation difficulty and duration (13).

Due to the acute angle of the left CCA origin in relation to the aortic arch, the anatomy of type III/bovine aortic arch adds difficulty in navigating the aortic arch for left-sided strokes (21). However, it was not statistically significant with FPE in this study. We speculate that it may be due to the aortic arch being the beginning of the vessel path. After the guidewire and catheter have passed through the aortic arch, it has little effect on whether the distal occlusion site can be recanalized for the first time.

A low CBS means that the thrombus is more widely obstructed, and we found that a thrombus with lower CBS was more difficult to remove through just one pass. Since EVT is a mechanical method of thrombus removal, the physical properties of the thrombus (e.g., length, volume) are critical to its technical success. In addition, a large clot burden often implies increments in volume and length. Hence, the friction between the thrombus and the vessel wall would be higher. Pulling the stent can lead to deformation and elongation of the vascular tissue, making the pathway longer and more difficult to manipulate (22). Previous studies have determined thrombus size on 3-dimensional CT images and found that patients with mFPE had significantly smaller thrombus volumes (23).

In our study, we observed that the truncal-type occlusion could achieve FPE much easier. We hypothesized that in the branching-site occlusions, it is difficult to remove both branches of the Y- or T- type occlusions through just one pass. In most instances, one of the branches can be removed in one operation at most, while the other branch would most likely break off in the vessel and require another attempt or more. Previous studies have correlated the angiographic occlusion type with the etiology. They found that truncal-type occlusion usually originated from atherosclerosis in large intracranial arteries, whereas branching-site occlusion is mostly due to cardiogenic embolism, lodged in the sharp turns of the bifurcation of a vessel (5, 11). One of the reasons of this is that cardiogenic thrombi seemed to have a higher proportion of fibrin compared to other stroke etiologies, making it associated with worse interventional recanalization (24). It was suggested that the atherosclerotic occlusion is more likely to cause intima injury, which would activate the platelets and again lead to occlusion. However, this situation is not very common and occurs after getting recanalization. Hence, this is not related to the rate of FPE in our study. In addition, for most patients with suspected intracranial arteriosclerosis, angioplasty or stent is the first choice in our center. Hence, these patients have been excluded.

Research showed that the rate of FPE would be increased by 3–4 times with the use of a balloon-guide catheter (8). The size of AC and the combined strategy (SR+AC) were also critical factors (25–27). We believe that the achievement of FPE would improve over time with experienced operators and new equipment and strategies specifically designed to improve clot removal efficiency.

Consistent with previous studies (8, 28), we also found that FPE can significantly improve the good prognosis of patients, regardless of mFPE and tFPE. The technically complete recanalization achieved after several passes is related to longer procedural time and poorer outcomes (20, 29), hence it being the so-called “futile” reperfusion. Furthermore, after multiple passes, the elastic morphology of thrombus changes, resulting in an increase in the sliding friction coefficient and make the clot become harder to retrieve (30). The future randomized clinical trials should pay more attention on how to provide a fast, complete, and safe revascularization during EVT.

## Limitations

Our study has several limitations. First, it was an observational study based on retrospective analysis. Hence, missing and unknown data might cause a selection bias. Second, this study included patients who were treated with SR, therefore, the interpretation of our results should be limited to the use of an SR. This is because the association between the thrombus characteristics and recanalization might differ among the mechanical devices used. Finally, this was a single-center, small sample-sized study. Therefore, a multicenter prospective study might be required in the future.

## Conclusion

In this study, the FPE rate was associated with CBS, the tortuosity of ICA, and the angiographic occlusion type. The rate of improved clinical and safety outcomes was higher in the FPE group compared to the non-FPE group. Therefore, achieving complete reperfusion at the first pass seems to be pivotal in the future of EVT treatment.

## Data Availability Statement

The raw data supporting the conclusions of this article will be made available by the authors, without undue reservation.

## Ethics Statement

The studies involving human participants were reviewed and approved by Scientific Research and New Technology IRB of Wannan Medical College Yijishan Hospital. The patients/participants provided their written informed consent to participate in this study. Written informed consent was obtained from the individual(s) for the publication of any potentially identifiable images or data included in this article.

## Author Contributions

CC, TZ, and XH designed the study. XX, JX, and KY contributed to data acquisition. YX, LY, and QY performed image analysis. CC and TZ wrote the primary manuscript. XH and ZZ contributed to critical revision and final approval of the manuscript. All authors contributed to the article and approved the submitted version.

## Funding

This work was supported by the National Natural Science Foundation of China (81171110), Wannan Medical College Foundation of Youths (WK2019F22), and Wannan Medical College Foundation of teaching quality and teaching reform (2020jyxm81).

## Conflict of Interest

The authors declare that the research was conducted in the absence of any commercial or financial relationships that could be construed as a potential conflict of interest.

## Publisher's Note

All claims expressed in this article are solely those of the authors and do not necessarily represent those of their affiliated organizations, or those of the publisher, the editors and the reviewers. Any product that may be evaluated in this article, or claim that may be made by its manufacturer, is not guaranteed or endorsed by the publisher.
